# Datasets of fingertip forces while grasping a handle with unsteady thumb platform

**DOI:** 10.1038/s41597-022-01497-x

**Published:** 2022-07-28

**Authors:** Banuvathy Rajakumar, Varadhan SKM

**Affiliations:** Department of Applied Mechanics, Indian Institute of Technology Madras, Chennai, India

**Keywords:** Motor control, Bone quality and biomechanics

## Abstract

This article presents the fingertip forces and moments data of the individual fingers and thumb when the thumb was placed on an unsteady platform, when the mass of the handle was systematically increased and when the thumb normal force was restricted while grasping a handle. Further, this article also includes a dataset while the thumb makes vertical movements such as extension (or upward motion) and flexion movement (or downward motion) during the static holding of a handle. An instrumented five-finger prehension handle was designed with a vertical railing on the thumb side. A slider platform was placed over the railing to mount the thumb force sensor. Further, a laser displacement sensor was mounted on top of the handle towards the thumb side to record the displacement of the thumb platform. The dataset includes fingertip forces, orientation of the handle, and the displacement data of thumb platform. This data helps therapists assess the degree of thumb disability, the contribution of ulnar fingers in establishing static equilibrium of a handheld object.

## Abstract (summary)

**Table:** 

Measurement(s)	Fingertip Forces and torques • Displacement • Orientation
Technology Type(s)	Strain gauge technology • laser technology • Electromagnetic Tracking Device Procedure
Factor Type(s)	Time
Sample Characteristic - Organism	Human participants

## Background & Summary

Grasping objects using hands is a common daily life activity. In the past, studies were performed to investigate individual fingertip force contribution when the grasped object undergoes systematic variations such as change in the mass^1,2^, external torques^3–5^, surface friction^6–8^, grip width^9^, individual digit width^10^ and fingertip position^4,11^. However, from the handles used in the current research, individual fingers force contribution was examined, especially ulnar fingers (ring and little), when the load contribution of the thumb was artificially reduced by placing the thumb on a slider platform. The platform was mounted over the vertical railing that was fitted on the thumb side of the grasping handle. None of the handles utilized in previous studies involved in understanding ulnar fingers contribution in stabilizing handle equilibrium when the load contribution of thumb was artificially reduced to a constant minimal magnitude.

In the current study, since the load contribution by the thumb was artificially reduced, it becomes the duty of the ring and little (ulnar) fingers normal forces to increase in order to compensate the role of thumb and sustain the vertical equilibrium of the handle^12^. Among the ulnar fingers, although little finger has a mechanical advantage (longer moment arm) than ring finger with respect to thumb as pivot point, it is smaller in structure compared to the ring finger. Therefore, in this research, we were curious to investigate the individual contribution of ulnar fingers in compensating the role of thumb. Whether the ring and little finger shares equally or little finger contributes greater than ring finger or vice versa at various conditions (or situations) was the general purpose of these experiments.

This article presents the dataset from four experiments. All the experiments were performed with the five-finger prehensile handle having an unsteady slider platform for the thumb. The first experiment was conducted as a preliminary study to gain basic understanding of how the ulnar fingers contribute in establishing the static equilibrium of the handle when the thumb was placed on a slider platform^12^. The second experiment involved in tracing patterns by displacing the thumb platform in the vertical direction^13^. The fingertip force data collected during this experiment helps to reveal the biomechanical relationship between thumb and peripheral fingers (index and little). The thumb displacement data collected during the upward (extension^14^) and downward (flexion) movement of the carpometacarpal joint of the thumb was fed to trace the pattern displayed on the monitor. The visual feedback of the thumb movement to the participant helps to regulate the movement efficiently. Thereby, it would result in speedy recovery of patients during the rehabilitation practice. Further, the third experiment was performed to investigate whether systematic variation in the mass of the handle influence the ulnar fingers force contribution. The fourth experiment was conducted to confirm whether task difficulty could influence the behaviour of the ring and little finger forces. The position and orientation data of the handle recorded during all the four experiments provided tilt feedback to the experimenter so as to validate whether the participant had performed the trial as per instruction.

This dataset enables us to understand finger mechanics by answering the following questions:

- how handle stabilization is achieved in the presence and absence of a mechanical constraint to fix the platform? (first experiment)
- how the contribution of individual fingers and thumb varies to achieve handle stabilization when there is any displacement of the thumb? (second experiment)
- how the finger force distribution pattern varies to maintain equilibrium when the mass of the handle is systematically increased with load force contribution of the thumb restricted to a constant minimal magnitude by placing on a slider platform? (third experiment)
- how handle stabilization is achieved when the thumb’s normal force is restricted to trace a solid horizontal target line that corresponds to a minimal normal force? (fourth experiment)

For all the trials of all the experiments, the participants were instructed to maintain the handle in static equilibrium. With a systematic variation in the mass of the handle or position of the thumb platform, residual pronation moment (anti-clockwise direction) would be noticed. In order to sustain the static equilibrium of the handle, participants had to voluntarily increase their normal forces of the ring and little fingers, producing a compensatory supination moment (clockwise direction) but not varying the tangential force of the thumb, as the thumb was placed on a slider platform.

The dataset collected from these experiments can be used as a baseline reference guide by the therapists in treating patients with ulnar nerve injury (especially male athletes) as a late phase rehabilitation therapy. By recording the forces exerted by the individual fingers of the patients while performing the grasping task, it is possible to quantitatively assess the functional recovery of the ring and little fingers strength. Compared to other studies, the dataset collected during these experiments helps to understand significant contribution of ulnar fingers in object stabilization when the load contribution of the thumb was artificially reduced. Also, the force data collected during the thumb movements helps researchers in designing ergonomic hand tool^15–17^.

## Methods

### Participants

Fifteen young healthy right-handed male volunteers in Experiment-1. Twelve right-handed males participated in Experiment-2. In Experiment-3, twelve young, healthy right-handed male volunteers participated. Twelve young healthy right-handed male participants volunteered to participate in the Experiment-4 (refer Table 1). All participants had no history of neurological disorders or trauma in the upper limb. All the participants signed the informed consent forms before the start of all the experiments. The experimental procedures were approved by the Institutional Ethics committee of the Indian Institute of Technology Madras (Approval number for Experiment 1: IEC/2016/02/VSK-2/12, Experiment 2: IEC/2018-03/SKM-2/05, Experiment 3 and 4: IEC/2021-01/SKM/02/05).

**Table 1:** Participant demographic details.

	Age (years)	Height (cm)	Weight (kg)	Hand-length (cm)	Hand-width (cm)
Experiment 1	25.6 ± 2.7	172.6 ± 3.9	73.3 ± 9.6	18.6 ± 0.9	8.7 ± 0.3
Experiment 2	22.6 ± 2.4	173.4 ± 6.4	70.5.3 ± 9.7	19 ± 0.6	9.5 ± 0.6
Experiment 3	26.75 ± 3.9	172.02 ± 5.7	75.21 ± 17.7	18.93 ± 1.1	8.92 ± 0.7
Experiment 4	26.66 ± 3.22	171.33 ± 7.54	76 ± 13.17	19.31 ± 0.70	9.02 ± 0.42

The table includes average age, height, weight, hand length and hand width of the participants with respective units. volunteered for all four experiments. The mean and standard deviation of the data are presented.

### Experimental setup

**Fig. 1:**
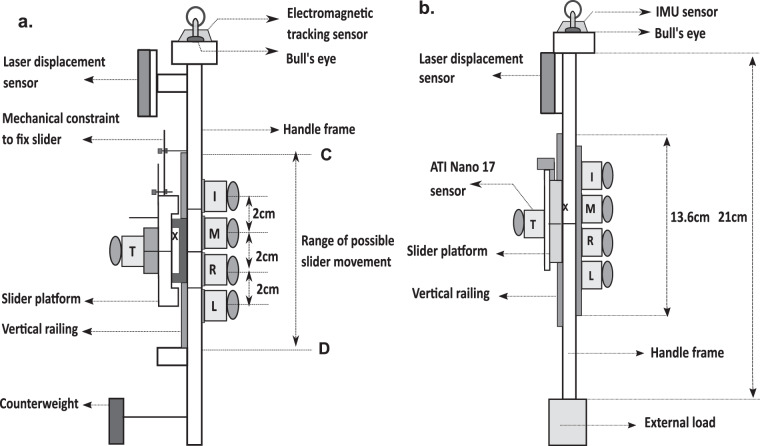
Schematic diagram of the experimental handles used for the prehension experiment. (**a**) Experimental handle utilized for experiments 1 and 2. The mass of the handle, including the counter-weight, was 0.535 kg. In the case of experiment 1, only two horizontal lines were drawn: one at the center of the thumb platform and the other at the midway between middle and ring fingers (HOME position). For experiment 2, in addition to the two horizontal lines, two more lines were drawn (1.5 cm above from HOME and 1.5 cm below from HOME). The dimensions of the handle frame of the handle used for the first and second experiment is (20 × 1 × 3) cm (**b**) Experimental handle utilized for experiment 3 and 4. The total mass of the handle, excluding the external load, was 0.450 kg. An external load of any of the mass 0.150 kg, 0.250 kg, 0.350 kg, and 0.450 kg was attached at the bottom of the handle below the center of mass of the handle (represented with symbol ‘X’) depending on the condition in the case of experiment 3. For both the conditions of experiment 4, an external load of mass 0.250 kg was attached at the bottom of the handle. The dimensions of the handle frame of the handle used for the third experiment are (21 × 1 × 3) cm. I, M, R, L, T represents index, middle, ring, little, and thumb.

**Fig. 2:**
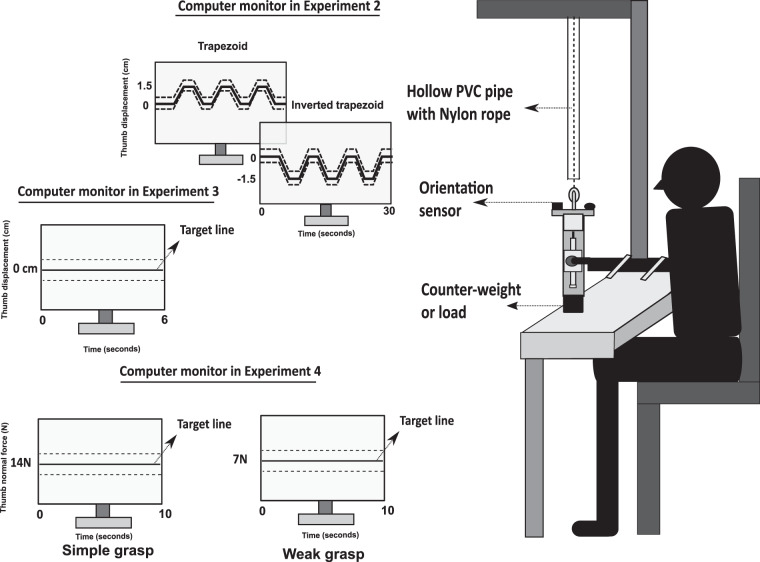
Schematic diagram of the experimental setup for the prehension experiment (**a**) The participant was holding the handle that was suspended from a wooden frame through a nylon wire which was housed within the hollow PVC pipe. An orientation sensor was placed a few cms above the forearm of the participant. For experiment 2, the computer monitor displayed the either a trapezoid or inverted trapezoid pattern depending on the condition. In the case of experiment 3, the computer monitor displayed solid horizontal target line with two dashed lines as acceptable error margins. In the fourth experiment, the participant’s monitor displayed solid horizontal target line that correspond to the normal force that need to be produced by the thumb. Depending on the experiment performed, handle was varied. For experiments 1 and 2, experimental handle with counterweight was used. Whereas for experiment 3 and 4, the handle with provision to suspend external load was used.

Two five-finger instrumented prehensile handles made of aluminum were designed and built by us for performing the experiments (refer Fig. 1). An experimental handle with counterweight was utilized for the first (simple grasping) and second (pattern tracing) experiments. Whereas a separate experimental handle without counterweight was used for the third (systematic variation of mass) and fourth (weak grasp) experiment. The entire mass of the handle (including the counterweight) utilized for experiments 1 and 2 was 0.535Kg. The total mass of the handle (excluding the external loads) used for experiment-3 was 0.450 kg. The handles were suspended from a wooden frame using nylon rope housed within the PVC pipe to restrict unnecessary movements of the handle (refer Fig. 2). Towards the thumb side of both the handles, a vertical railing was provided over which a slider platform of mass 0.101Kg was placed. The slider platform could translate on the entire length of the railing as the friction between the platform and railing was kept minimal by regularly cleaning and lubricating the ball bearings in the slider. Five six-component force/torque sensors (Nano 17, Force resolution: Tangential: 0.0125 N, Normal: 0.0125 N, ATI Industrial Automation, NC, USA) were mounted on the grasping handle to measure the forces and moments of individual fingers and thumb. The force sensor to measure the thumb’s force was mounted on the slider platform, while the other force sensors were placed on the side opposite to the railing.

An acrylic block was placed in the anterior-posterior direction on the top of the handle to mount the electromagnetic tracking sensor (Resolution 1.27 microns, Static position accuracy 0.76 mm, Static angular orientation accuracy 0.15°, Model: Liberty Standard sensor, Polhemus Inc., USA) for experiments 1 and 2. It gives the position and orientation data of the handle with respect to the transmitter. For experiment 3 and 4, an IMU (Inertial measurement unit) sensor (Resolution: 16bits, Range: 2000°/s, Model: BNO055, BOSCH, Germany) was mounted on the acrylic block to measure the orientation of the handle after appropriate pre-processing of the raw data. On the participant’s side of the acrylic block of both the handles, a spirit level with the bull’s eye was placed for the participant to monitor the tilt caused in the handle.

For both the handles, the laser displacement sensor (resolution, 5μm; OADM 12U6460, Baumer, India) was mounted over the flat acrylic protrusion placed on the thumb side of the handle to measure the displacement of the thumb in the vertical direction. The same experimental handle with counterweight was used for both experiments 1 and 2. An aluminum counterweight of mass 0.035Kg was attached at the bottom of the handle towards the thumb side in order to shift the center of mass close to the geometric center of the handle. However, in the case of the handle used for experiment 3, counterweight was not used. Instead, external loads of masses 0.150 kg, 0.250 kg, 0.350 kg, and 0.450 kg were used at the bottom of the handle. For the fourth experiment, an external load of 0.250 kg was attached at the bottom of the handle of mass 0.450 kg.

**Fig. 3:**
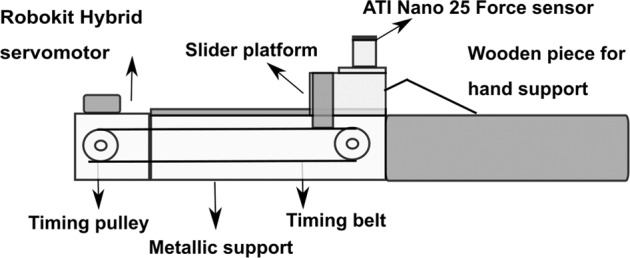
Side view of the friction setup showing force sensor, timing pulleys, and belt. A force sensor is mounted on top of the slider platform that translates in the vertical direction. In this figure, the slider platform is at its start position.

For fingertip friction measurement, a separate device was built with the provision to mount a six-component force/torque sensor (Nano 25, ATI Industrial Automation, Garner, N.C) on the top of the aluminum platform, which moved in the horizontal direction over a railing fitted on rectangular metallic support (shown in Fig. 3). The platform moved linearly with the help of a timing belt-pulley system powered by a servomotor^18,19^. A customized LabVIEW program was written for the force/torque data collection and to control the operation of the motor. Velcro straps arrested the forearm and wrist movements of the participants while a wooden block was placed underneath the participant’s palm for the steady hand and finger configuration.

### Experimental procedure

The participants were instructed to lift the handle (approximately 2 cm) upwards from the suspended position before the start of the trial of all the prehension experiments in order to have zero force in the suspension cable.

### Prehension Experiment-1

**Fig. 4:**
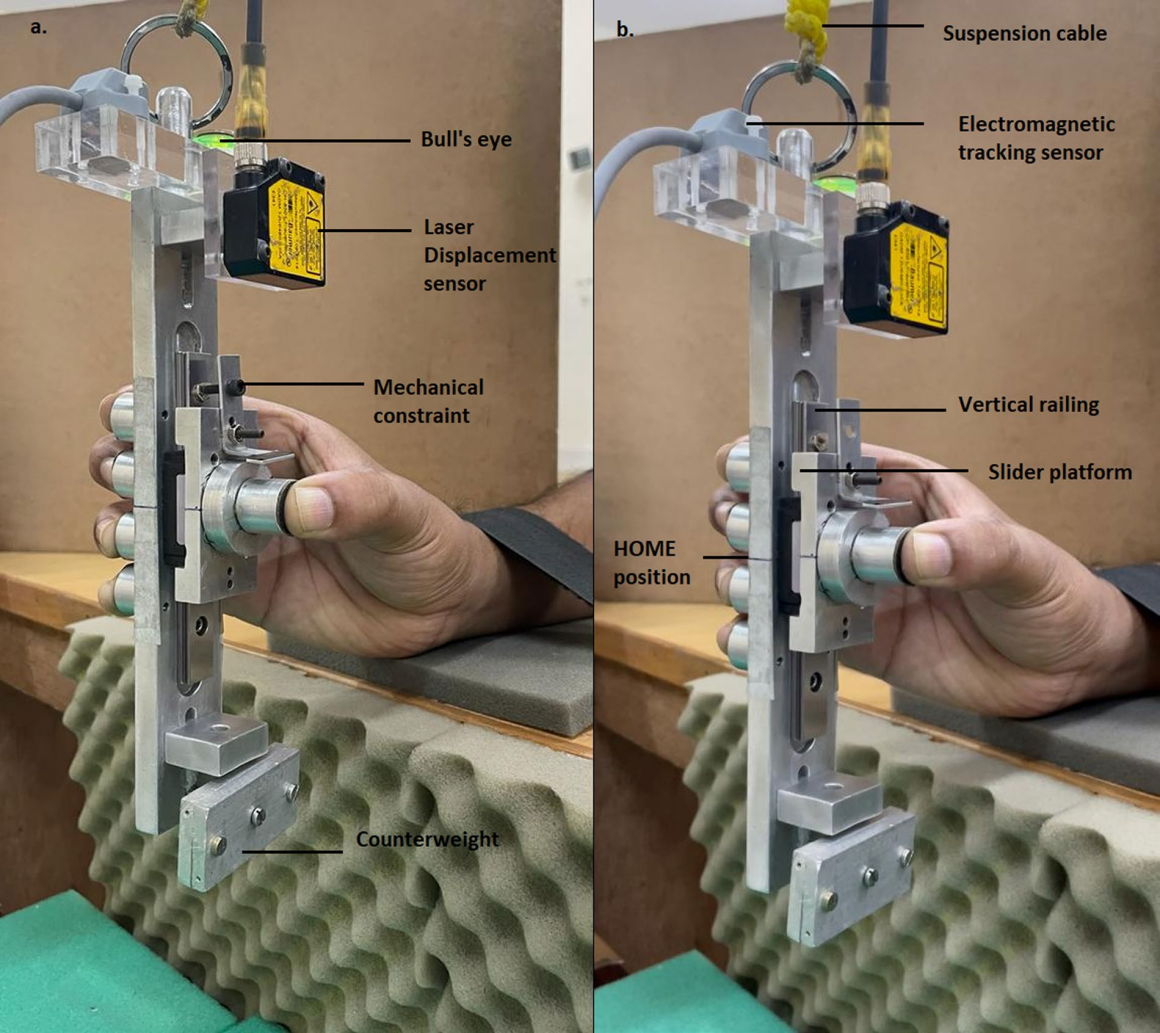
The experimental handle with counterweight utilized for first and second experiment (**a**) Left panel: The experimental handle when used for the ‘fixed’ condition of the first experiment. The mechanical constraint was fitted to arrest the movement of slider platform. (**b**) Right panel: The same experimental handle used for the ‘free’ condition of the first experiment. The mechanical constraint was removed and therefore, the slider could translate in the vertical railing using thumb. For the first experiment, two horizontal lines were drawn on the participant’s side of the handle: one at the center of slider platform and another line drawn midway between middle and ring finger’s sensor center on the handle frame. For the sake of visualizing, the horizontal lines were drawn on the other side of the participant. Since the force sensors were mounted on a different handle for a different experiment, dummy force sensors were mounted. The photograph was captured by the first author Banuvathy Rajakumar and the participant is the other author Dr Varadhan SKM.

The first experiment consisted of two conditions: ‘fixed’ and ‘free’ condition. In the fixed condition, a mechanical constraint (see Fig. 4a) was used to arrest the movement of the slider platform. On the participant’s side of the handle, two horizontal lines were drawn. One horizontal line was drawn at the center of the slider platform, and another line was drawn on the handle frame at the midway of the middle and ring finger’s sensor center (referred as HOME position). Before the beginning of the trials in the fixed condition, the two horizontal lines were matched and the movement of the slider platform was arrested using the mechanical constraint. Although the fixed condition was a simple grasping of a handle similar to other studies, it was performed so as to compare with fingertip forces data collected from the free condition.

In the free condition, the mechanical constraint was removed and therefore the platform could be translated in the vertical direction using the thumb. However, the instruction was to hold the slider platform steady by aligning the horizontal line drawn on the platform to the line drawn midway between the middle and ring fingers with the help of the thumb (refer Fig. 4b).

During both the conditions, the task was to maintain the handle in static equilibrium throughout the trial. It was ensured by the participants by placing the bubble at the center of the bull’s eye.

### Prehension Experiment-2

**Fig. 5:**
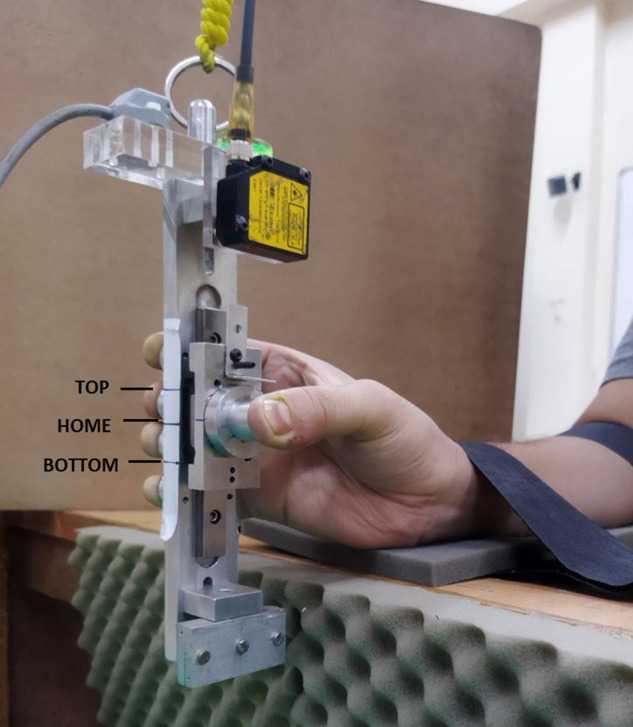
Experimental handle used for the second experiment with mechanical constraint removed. In addition to the two horizontal lines (one on the platform and another on the handle frame), two additional horizontal lines were drawn on the handle frame. At 1.5 cm above and below the line drawn at the HOME position, two additional horizontal lines were drawn signifying TOP and BOTTOM position. The mechanical constraint was removed so that the slider could translate over the railing and complete performing the tracing task. The photograph was captured by the first author Banuvathy Rajakumar and the participant was the other author Dr Varadhan SKM.

The second experiment was a tracing task and consisted of two conditions: ‘trapezoid’ and ‘inverted trapezoid’ condition. The same experimental handle (see Figs. 1a and 5) that was used for the first experiment was used for the second experiment. However, there was no mechanical constraint used to fix the slider platform for both the conditions of the second experiment. Therefore, the slider platform could translate freely in the vertical direction using thumb. The thumb displacement data was shown as a visual feedback line to trace the pattern displayed. During both the conditions, the task was to trace the pattern displayed on the monitor by displacing the slider platform using the thumb in the vertical direction. The thumb displacement data was shown as a visual feedback line to the participant to trace the pattern shown on the monitor^13^.

**Fig. 6:**
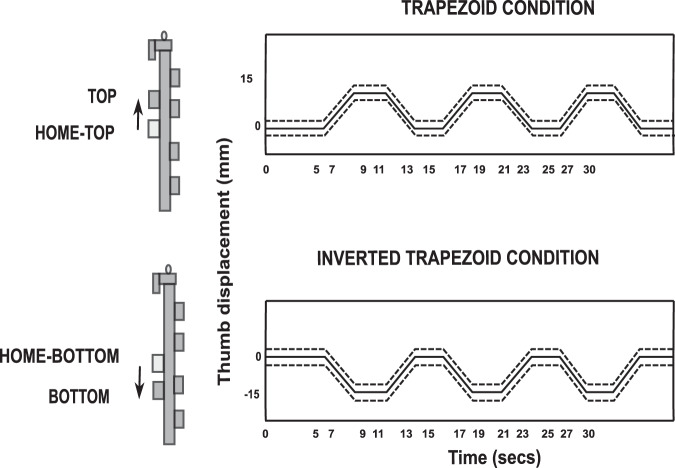
Diagram shows four static positions of the thumb and the patterns displayed on the computer monitor during each condition. The figure shows the static TOP (dark shaded) and HOME-TOP (light shaded) positions of the thumb during the trapezoid condition (top panel). Also, the static BOTTOM (dark shaded) and HOME-BOTTOM (light shaded) positions of the thumb during inverted trapezoid condition (bottom panel) were shown.

In a trial of trapezoid condition, three trapezoid patterns were arranged consecutively with a static ‘flat’ portion of two seconds between two trapezoids (see Fig. 6). For the first five seconds of the trial, the participants were instructed to place the slider platform at the HOME position (matching the horizontal line on the platform to the line drawn midway between middle and ring fingers). Thereby, tracing the initial static ‘flat’ portion before starting the first trapezoid pattern (refer Fig. 6). Then, the slider platform was gradually translated 1.5 cm upwards by tracing the ‘ramp’ portion of the first trapezoid. At the new TOP position (indicated by horizontal line drawn at 1.5 cm above HOME position on the participant’s side of the handle frame), the slider was held steady for the next two seconds by aligning the line on the thumb platform to the horizontal line drawn 1.5 cm above the HOME position. This was then be followed by tracing the second ‘ramp’ portion of the same trapezoid pattern. Likewise, the participants traced the remaining two trapezoid patterns to complete the trial. The static HOME position when reached by translating slider platform from TOP position during trapezoid condition was referred as HOME-TOP position (refer Fig. 6).

In the inverted trapezoid condition, within a trial, three inverted trapezoid patterns were arranged consecutively with a static ‘flat’ portion of two seconds between two successive patterns. For the first five seconds, the initial static ‘flat’ portion of the trial in the inverted trapezoid condition was traced by aligning the horizontal line at the center of the thumb platform to the line drawn midway between middle and ring fingers on the handle frame. The participants were then required to translate the slider platform 1.5 cm downwards by tracing the ‘ramp’ portion of the inverted trapezoid pattern (refer Fig. 6). At the new BOTTOM position (indicated by horizontal line drawn at 1.5 cm below HOME position on the participant’s side of the handle frame), the slider was held steady for the next two seconds. This was ensured by matching the line on the thumb platform to the horizontal line drawn 1.5 cm below the HOME position. Thus, this was then be followed by tracing the second ‘ramp’ portion of the same inverted trapezoid pattern. Similarly, the participants had to trace the remaining two inverted trapezoid patterns to complete the trial. The static HOME position when reached by translating slider platform from BOTTOM position during inverted trapezoid condition was referred as HOME-BOTTOM position (refer Fig. 6).

At the end of the ramp portion of the third pattern of each trial in both the conditions, the slider platform was held steady at the HOME position by tracing the final static ‘flat’ portion for three seconds. This was accomplished by aligning the thumb platform line to the line on the handle frame (HOME position). And thus, the end of the trial would be reached.

While tracing the pattern displayed on the monitor, i.e, during the entire trial of both the conditions (trapezoid and inverted trapezoid), the handle had to be maintained in static equilibrium. The static equilibrium of the handle was ensured by the participants by placing the bubble at the center of the bull’s eye during the trial.

### Prehension Experiment-3

**Fig. 7:**
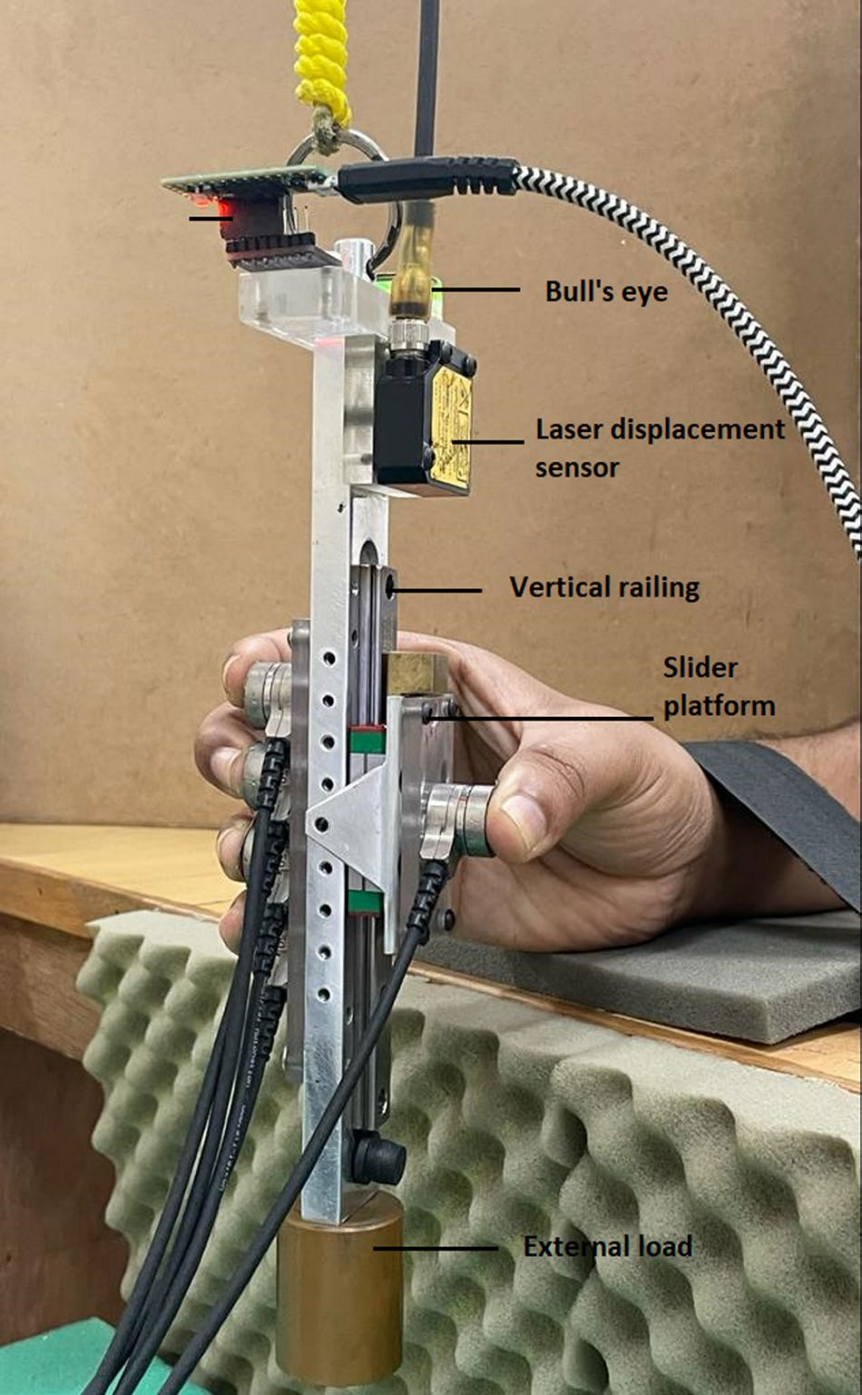
Experimental handle utilized for the third and fourth experiments. Instead of a counterweight, a provision was provided at the bottom of the handle to attach external loads of varying masses. In the case of the third experiment, external loads of different masses (0.150 kg, 0.250 kg, 0.350 kg, 0.450 kg) was attached. For both the conditions of the fourth experiment, same external load of 0.250 kg was attached. The photograph was captured by the first author Banuvathy Rajakumar and the participant was the other author Dr Varadhan SKM.

The third experiment was comprised of four conditions. The other experimental handle (see Figs. 1b and 7) without counterweight was used for the third experiment. Similar to the experimental handle used for the first experiment, two horizontal lines were drawn on the participant’s side of the other handle also. And mechanical constraint was not provided with the handle used for the third experiment. Therefore, the platform could translate over the vertical railing using thumb.

In each condition of the third experiment, the external load of mass 0.150 kg, 0.250 kg, 0.350 kg, and 0.450 kg was added at the bottom of the handle below the center of mass^20^. The instruction was to hold the thumb platform steady at the HOME position by aligning the two horizontal lines closely. Thumb displacement data was shown as visual feedback line from the beginning of the trial to trace the solid horizontal target line displayed on the monitor (see Video 8 at Figshare). The target line displayed on the monitor corresponds to thumb’s HOME position. Two dashed lines drawn at 0.2 cm above and below the solid target line on the monitor was the error margins. A successful trial was ensured by matching the thumb displacement feedback line to the target line within the acceptable error margin of ±0.2 cm. Further, the participants were instructed to maintain the static equilibrium of the handle by positioning the bubble at the center of the spirit level throughout the trial when an external load was attached at the bottom of the handle during each condition.

### Prehension Experiment-4

The fourth experiment consisted of two conditions: simple grasp and weak grasp condition. The experimental handle (see Figs. 1b and 7) utilized for third experiment was used for the fourth experiment also. For both simple and weak grasp conditions, an external load of 0.250 kg was attached at the bottom of the handle.

The computer monitor displayed a solid horizontal target line that corresponds to thumb normal force (i.e,) 14 N (in the case of simple grasp condition) or 7 N (in the case of weak grasp condition). The normal force produced by the thumb was shown as visual feedback line to trace the target line (see Video 9at Figshare). The task was to produce thumb normal force matching to the solid horizontal target line within the acceptable error margin of ±0.5 N which were represented in the form of dashed lines above and below the target line. The mechanical constraint to arrest the movement of slider platform was not provided for the handle used for the fourth experiment. Therefore, the slider platform could translate in the vertical direction using the thumb.

However, an instructed amount of thumb normal force had to be produced by holding the platform steady at the HOME position. This was ensured by aligning the horizontal line drawn at the center of the platform to the horizontal line drawn midway between middle and ring fingers on the handle frame by the participants. Throughout the trial, during both the conditions, static equilibrium of the handle had to be maintained by positioning the bubble at the center of bull’s eye. An acceptable error margin for the thumb displacement data was ±0.2 cm. The experimenter could view the thumb displacement data, net tilt angle, normal and tangential forces of all fingers and thumb during the trial in a separate monitor.

### Friction experiment

Friction experiments were conducted at the beginning of all four prehension experiments. It involved one trial per finger (experiment 1) and two trials per finger (experiment 2, 3 and 4), with a one-minute break provided between the trials. The force/torque data from the thumb and index finger were collected. With the tangential and normal force data collected from the instructed finger, friction coefficient was computed. By using the friction coefficient values of the finger and thumb, safety margin analysis can be performed.

During the friction experiment, the participants were instructed to produce a constant downward normal force of 6 N for 3 s to initiate movement of the servomotor. Visual feedback of the normal force produced by the instructed finger was shown on the computer monitor for the participant. The platform moved at a speed of 6 mm/s away from the participant.

### Experimental protocol

For the current research, repeated measures design has been adopted in designing all four experiments. According to this design method, multiple measures were taken for the same variable, and same set of participants under different conditions. By having a greater number of trials (repeated measures) with limited number of participants, a better statistical power can be achieved. As a result, if there exists any effect, the statistical test that we employ can detect the effect accurately.

Since our first experiment was conducted as a preliminary study, we considered having a maximum number of 30 trials for each condition, in order to attain a high statistical power and to ensure if the participants were able to perform the trials without experiencing any fatigue. From the results on the analysis performed with the pilot data collected, we could find that the high statistical power with no fatigue can be achieved with maximum of 30 trials for each condition of the first experiment. Therefore, each condition of the first experiment consisted of thirty trials. The trial duration was 10 seconds (as it was a preliminary grasping study with only two conditions). A one-hour rest period was provided between conditions. Eight participants performed the free condition first, followed by the fixed condition, while seven participants performed the fixed condition first, followed by the free condition.

In the case of second experiment, each trial lasted for 30 seconds. The second experiment involved two conditions: trapezoid and inverted trapezoid condition. In each condition, there were twelve trials. Since our focus was to analyse the individual fingers force data at different static positions (TOP, HOME-TOP, HOME-BOTTOM, BOTTOM) of the thumb, three trapezoid patterns or inverted patterns were accommodated for single trial. Therefore, in each trial of trapezoid condition, three static TOP and HOME-TOP positions were seen. While, in case of inverted trapezoid condition, three static HOME-BOTTOM and BOTTOM positions were present. With twelve trials in each condition, totally 36 ‘flat’ segments for each static position were considered for analysis. The time duration of each segment was two seconds. Thus, to accommodate three consecutive trapezoid patterns in a trial, total trial duration was set to 30 seconds. Although ten trials are enough to have maximum of 30 segments for each static position, 12 trials were collected from each participant to have a better statistical power. A rest period of at least one-minute was provided between the trials. Six participants performed trapezoid condition first, followed by an inverted trapezoid condition. The other six participants performed inverted trapezoid condition first, followed by trapezoid condition. A break of 30 minutes was provided between the conditions. The entire experiment was completed within two hours.

The third experiment was conducted as two separate sessions. In each session, two external loads were included with thirty minutes of break between conditions. For each external load condition, 25 trials were provided. Since there exist four different conditions with the addition of an external load for each condition, number of trials were reduced to 25 in order to avoid the effect of fatigue and to balance the trials across the conditions. For the same reason, the duration of each trial was also brought down to six seconds. One minute break was provided between trials. After every twelve trials of each condition, ten minutes break was provided. The order of presentation of these sessions was counterbalanced across all participants. Six participants performed the external load condition of 0.150 kg followed by 0.350 kg in their first session. The other six participants performed the external load condition of 0.450 kg followed by 0.250 kg in their first session.

Although the fourth prehension experiment consisted of only two conditions (simple and weak), the task of exerting minimal amount of thumb normal force (weak grasp condition) while holding the slider steady at HOME position was quiet challenging. Therefore, to complete the challenging task successfully and to avoid the effect fatigue while performing the task, only 25 trials were provided for each condition. In addition to this, since the task of producing required amount of thumb normal force exactly matching to the target line takes time with different participants and trials, the trial duration was extended to 10 s. One minute rest was provided between the trials and half an hour break was provided between the conditions. Six participants performed weak grasp first, followed by the simple grasp condition. The other six participants performed simple grasp condition first, followed by weak grasp condition.

The experimental protocol details of all four experiments are shown in Table 2.

**Table 2:** Experimental protocol details of all four experiments.

Experiment No.	No. of participants	No. of conditions	No. of trials per condition	Duration of each trial (seconds)
Experiment 1	15	2	30	10
Experiment 2	12	2	12	30
Experiment 3	12	4	25	6
Experiment 4	12	2	25	10

Table shows the number of the participants participated and number of conditions in each experiment. Also, the number of trials per condition also shown with duration of each trial.

### Experimental preparation

The participants washed their hands and towel-dried once after entering the experimental room. After the completion of the experiment by each participant, a new set of sandpaper (100 grit sandpaper) was used on the force sensors surface of the handle and friction setup. Before initiating the data collection process, the instructions to perform the task was explained verbally by the experimenter to the participant. After explaining the procedure to perform the task, the experimenter demonstrated a trial from each condition for the participant. The participants were also provided with a few familiarization trials to make them feel comfortable before performing the main experiment. These trials were not recorded.

Following were the instructions read out to the participants by the experimenter before commencing the experiment.

- Please do not overgrip the sensors.
- Please place the center of the fingerpad of each finger to the force sensor’s center and then lift the handle approximately 2cms from the suspended position.
- Before starting each trial, hold the handle vertical to maintain the bubble in the center of the bull’s eye.
- Inform the experimenter once after positioning the bubble at the center of the bull’s eye.
- Please maintain the handle in static equilibrium throughout the trial during all conditions by keeping the bubble at the center of the bull’s eye.
- Please ensure whether the thumb platform is positioned comfortably at the HOME position by matching the horizontal line on the thumb platform to the horizontal line drawn between the middle and ring finger sensor of the handle (which was considered as HOME position) before the start of the trial.
- For the second experiment: Please trace the patterns within the acceptable error margin of ± 0.5 cm by displacing the thumb from the HOME position to 1.5cms upwards and then back to the HOME position during trapezoid condition. Similarly, during the inverted trapezoid condition, move the thumb platform from the HOME position to 1.5 cms downward and then back to the initial position. In the case of the third experiment: Please trace the solid horizontal target line shown on the monitor with the thumb displacement feedback line by aligning the two horizontal lines on the thumb platform and handle frame.
- For the fourth experiment, during weak grasp condition, please grasp the handle with minimal grip force so that the feedback line of the thumb normal force matches the solid horizontal target force line shown on the participant’s monitor. During simple grasp condition, please produce thumb normal force matching to the solid target line displayed on the participant’s monitor.
- Please inform the experimenter when you feel any discomfort. You will be provided rest as needed.

### Data processing

Thirty analog signals from the force/torque sensors (5 sensors x 6 components) and single-channel analog laser displacement data were digitized using NI USB 6225 and 6002 at 16-bit resolution (National Instruments, Austin, TX, USA). Force/torque and displacement data were synchronized with six channels of processed, digital data from the electromagnetic tracker for experiments 1 & 2 and four channels of processed quaternion data from experiment-3 & 4. The force signals from the force sensors and orientation signals from polhemus sensors were synchronized in LabVIEW software using predefined library files within a same data collection loop. In particular, both the signals were collected at same sampling frequency within a same data collection loop. The dataset was collected at a sampling frequency of 100 Hz. The raw data were imported to Matlab for further processing. The data were filtered in Matlab through the second-order zero phase lag Butterworth low pass filter (cut off frequency, 15 Hz) for the prehension and friction experiments. In each experiment, data was selected from specific time interval for the computation of the below mentioned forces and moments. In the case of the first experiment, data from 2.5 s to 7.5 s of each trial were selected. While, for the second experiment, the first one second data from the static positions (TOP, HOME-TOP, HOME-BOTTOM, BOTTOM) were considered. With regard to the third experiment, data from 2 s to 5 s of each trial was taken. In the case of fourth experiment, data from 3 s to 7 s of each trial was taken for forces, and other computations.

The main reason for performing computation using the datapoints within the particular time window in the experiments was to eliminate start and end effects of a trial. With regard to the second experiment, since the main focus was to analyse the individual fingers force data when the thumb was held at different static positions (TOP, HOME, BOTTOM), only the first one second data (100 datapoints) was chosen from the three static positions of the thumb for analysis. Perhaps, even if an entire two seconds data at the three different static positions of the thumb was considered for analysis, the results would not be significantly different.

### Code description

#### forceanddisp_dataplots.m

This Matlab code consists of four sections. Each section corresponds to an experiment. Each section obtains data from the respective folder and filters the individual fingertip forces and displacement data. After filtering, the normal (grip) and tangential (load) forces of all the fingers would be averaged across trials and participants. The averaged time series plot of the individual finger’s normal forces, tangential forces, and thumb displacement data of all the conditions of all the experiments would be displayed separately as figures.

#### momentcomputation.m

This Matlab code is a sample code to compute moments exerted by individual fingers and thumb during the fixed and free conditions of the first experiment. The center of pressure of all the fingers and thumb were computed and accounted for the computation of moment arms of the normal forces.

#### filter_code.m

This Matlab code is responsible for filtering the forces and thumb displacement data. After fetching the data, this code involves in the filtering of the data.

## Data Records

The raw data along with the code can be found at figshare^21^.

Figshare link: 10.6084/m9.figshare.19207875.

The first experiment on a simple five-finger grasping task was collected from fifteen participants. The data from each participant was stored in fifteen separate folders. Within each participant folder, there exist two subfolders: fixed condition and free condition. Within the condition subfolder, thirty data files in .csv format would be present. Each column contains 1001 rows (sampling frequency = 100 Hz, single-trial duration = 10 seconds).

The second experiment on the five-finger grasping task that involves tracing the pattern by displacing the thumb was collected from twelve participants. The data from each participant were stored in twelve separate folders. Within each folder, two subfolders were created for two different conditions, which were named trapezoid and inverted trapezoid conditions. Twelve data files (in .csv format) are present in each subfolder. Each column of the data file contains 3001 rows.

The data of the third experiment on the five-finger grasping task that involves a systematic increase in the mass of the handle of twelve participants were stored in twelve separate folders. Within each folder, four subfolders were created for four different conditions, which were named as onefifty, twofifty, threefifty, and fourfifty. Twenty-five data files (in .csv format) are present in each subfolder. Each column of the data file contains 601 rows.

The fourth experiment data was stored in twelve separate folders. Within each folder, four subfolders were created for two different conditions, which were named as simplegrasp and weakgrasp. Similar to the third experiment, twenty-five data files (in .csv format) are present in each subfolder. Each column of the data file contains 1001 rows.

Each data file contains data from a single trial. For experiments 1 and 2, thirty-eight columns of data were present for each trial (refer to Table 3). The first thirty columns indicate force and torque data from index, middle, ring, little, and thumb. They were followed by six columns of position (X, Y, Z) and orientation (Azimuth (A), Elevation (E), Roll (R)) data of the handle. The thirty-seventh column indicates the thumb displacement data. The last column signifies the Polhemus data availability flag.

**Table 3:** Data in each column of experiments 1 and 2 are shown in this table.

Column 1 to 30					Column 31 to 36	Column 37	Column 38
Index	Middle	Ring	Little	Thumb	Position (cm) and orientation (°) data of handle	Thumb displacement data (Experiment 1 in mm, Experiment 2 in cm)	Polhemus data availability flag
Force (Fx, Fy, Fz) & torque (Mx, My, Mz)	Force (Fx, Fy, Fz) & torque (Mx, My, Mz)	Force (Fx, Fy, Fz) & torque (Mx, My, Mz)	Force (Fx, Fy, Fz) & torque (Mx, My, Mz)	Force (Fy, Fx, Fz) & torque (My, Mx, Mz)	X, Y, Z, A, E, R		0 or 1

The table shows the column-wise data arrangement of forces (N), torques (Nmm) in all three directions of each finger, position and orientation data of the handle and thumb displacement data in the datafile.

Whereas, for experiment 3 and 4, thirty-six columns of data were present for each trial (refer to Table 4). The first thirty columns indicate force and torque data from index, middle, ring, little, and thumb. They were followed by four columns of orientation data of the handle in the form of quaternions (w, x, y, z). The thirty-fifth column indicates the net tilt angle data computed directly from the raw quaternion data. The last column signifies the thumb displacement data.

**Table 4:** Data in each column of experiment 3 and 4 are shown in this table.

Column 1 to 30					Column 31 to 34	Column 35	Column 36
Index	Middle	Ring	Little	Thumb	Orientation data of handle (Quaternions)	Net tilt angle (degrees)	Thumb displacement data (cm)
Force (Fx, Fy, Fz) & torque (Mx, My, Mz)	Force (Fx, Fy, Fz) & torque (Mx, My, Mz)	Force (Fx, Fy, Fz) & torque (Mx, My, Mz)	Force (Fx, Fy, Fz) & torque (Mx, My, Mz)	Force (Fy, Fx, Fz) & torque (My, Mx, Mz)	w, x, y, z	Ѳ	

The table shows the column-wise data arrangement of forces (N), torques (Nmm) in all three directions of each finger, orientation data of the handle, net tilt angle, thumb displacement data in the datafile.

### Description of the data columns from 1 to 30 of all four experiments

First six columns (column number: 1,2,3,4,5,6) includes forces (N) and moments (Nmm) data of Index finger: Fx(horizontal tangential force), Fy (vertical tangential force), Fz (normal force), Mx(Moment about horizontal tangential force), My(Moment about the vertical tangential force), Mz(Moment about the normal force)

The second set of six columns (column number: 7,8,9,10,11,12) includes forces (N) and moments (Nmm) data of the Middle finger: Fx, Fy, Fz, Mx, My, Mz.

The third set of six columns (column number: 13,14,15,16,17,18) includes forces (N) and moments (Nmm) data of Ring finger: Fx, Fy, Fz, Mx, My, Mz.

Fourth set of six columns (column number: 19,20,21,22,23,24) includes forces (in N) and moments (in Nmm) data of Little finger: Fx, Fy, Fz, Mx, My, Mz.

Fifth set of six columns (column number: 25,26,27,28,29,30) includes forces (in N) and moments (in Nmm) data of Thumb: Fy (vertical tangential force), Fx (horizontal tangential force), Fz (normal force), My, Mx, Mz. Note: Here, the vertical tangential force of thumb belongs to 25^th^ column of data due to the change in the orientation of the force sensor of the thumb

### Description of the columns from 31 to 38 of the first two experiments

The sixth set of six columns (column number: 31,32,33,34,35,36) includes position (cm) and orientation (degrees) data of the handle: X (vertical distance between the origin of the source and the sensor at the handle), Y(horizontal distance between the origin of the source and the sensor at the handle in front-back direction), Z (horizontal distance between the origin of the source and the sensor at the handle in the left-right direction), Azimuth (Ѳx), Elevation (Ѳy), and Roll (Ѳz).

The thirty-seventh column of the dataset consists of the thumb displacement data measured from the laser displacement sensor. For the first experiment, the unit of thumb displacement data was in millimeters (mm). Whereas, for the second experiment, the unit of thumb displacement data was in centimeters (cm).

The last column of data is the ‘Polhemus data availability flag’. It would be either 1 or 0. In the absence of position data at any particular instance during the trial will have 0.

### Description of the columns from 31 to 36 of the third and fourth experiment

The sixth set of four columns (column number: 31, 32, 33, 34) includes orientation data of the handle in the form of quaternions (w, x, y, z).

The thirty-fifth column of the dataset consists of the net tilt angle data computed directly from the raw quaternions data.

The last column of data is the thumb displacement data (cm) measured from the laser displacement sensor.

Depending on the experiment, each CSV file was named in the following nomenclature: ‘(Participant ID)_trial_(trial no)_(condition name).csv.’

### Description of the data columns of the Friction experiment

First six columns (column number: 1,2,3,4,5,6) includes forces (in N) and moments (in Nmm) data of the finger (either index or thumb): Fx(horizontal tangential force), Fy (vertical tangential force), Fz (normal force), Mx(Moment about horizontal tangential force), My(Moment about the vertical tangential force), Mz(Moment about the normal force)

## Technical Validation

- **Position and Orientation data validation**
  The entire experimental handle, counter-weight, and the experimental table were made from material like aluminum and wood in order to get rid of the magnetic interference caused due to the usage of the electromagnetic tracker. Further, the electromagnetic tracking sensors were properly calibrated prior to the data collection. Besides all this, before the start of the trial, a command called *Boresight (ctrl* + *B)* was used to set the initial position and orientation of Polhemus data as zero with reference to the electromagnetic transmitter. Therefore, during the trial, at any instance, a change in the position or orientation of the handle from its initial configuration would be measured accurately. From the orientation data such as azimuth, elevation, and roll data (in degrees), the net tilt angle was computed for the first and second experiments. As a preliminary step, the orientation data were low pass filtered with a cut-off frequency of 25 Hz and converted to radians. Next, the filtered angular data was used to construct direction cosine matrix (or rotation matrix). For each rotation matrix, the trace (sum of the diagonal elements) of the matrix was calculated. Finally, the net tilt angle data (Ѳ) was computed using the following Eq. (1) and displayed in the experimenter monitor. Net tilt angle in the form of time series was computed for each trial.
  1 $${\rm{\theta }}{=\cos }^{-1}\left[\frac{trace-1}{2}\right]$$
  In the case of the third and fourth experiment, the net tilt angle was directly computed from the raw quaternions and stored separately in the 35^th^ column of the data file.
  Finally, the net tilt angle was then averaged across the trials, samples, and participants. Table 5 shows the average net tilt angle during the four different experiments. The first 2 s data were averaged and then subtracted from each sample of Ѳ of the trial to remove any baseline shift.

**Table 5:** Average Net tilt angle with the standard deviation of all the conditions during the four experiments are shown. SD represents standard deviation.

Outcome variable	Experiment	Condition	Thumb position	Mean (°)	SD
Net tilt angle	Experiment 1	Fixed	Home	4.13	2.31
		Free	Home	3.83	1.82
	Experiment 2	Trapezoid	Top	1.29	0.47
			Home-Top	1.40	0.44
		Inverted Trapezoid	Home-Bottom	2.13	1.05
			Bottom	2.06	0.83
	Experiment 3	0.150 kg	Home	0.58	0.22
		0.250 kg	Home	0.73	0.19
		0.350 kg	Home	0.70	0.15
		0.450 kg	Home	0.81	0.23
	Experiment 4	simple grasp	Home	0.72	0.24
		weak grasp	Home	0.78	0.25
- **Force data validation**

The instruction to the participants was to maintain the handle in static equilibrium throughout the trial. To achieve static equilibrium, the following criteria have to be satisfied.

- The sum of the normal forces of the index, middle, ring, and little fingers should be equal to the normal force of the thumb. Table 6 shows the average normal force produced by each finger and thumb during the handle stabilization during all four experiments. It was found that the sum of normal forces produced by the index, middle, ring, and little fingers was close to the normal force produced by the thumb.

**Table 6:** Average normal force data of the individual fingers and thumb during different conditions of the four experiments are shown.

Outcome variable	Condition	Thumb position	Index	Middle	Ring	Little	Thumb	I + M + R + L
**Normal force**	**Fixed**	**Home**	1.63	1.33	1.21	1.09	5.36	5.26
	**Free**	**Home**	1.65	1.84	2.69	2.86	9.16	9.04
	**Trapezoid**	**Top**	3.51	1.96	1.89	1.22	8.70	8.58
		**Home-Top**	1.64	1.79	2.59	3.43	9.58	9.45
	**Inverted Trapezoid**	**Home-Bottom**	2.15	1.86	2.90	2.78	9.79	9.69
		**Bottom**	0.80	0.94	3.02	7.25	12.13	12.01
	**0.150** **kg**	**Home**	1.79	2.58	4.55	4.75	13.73	13.67
	**0.250** **kg**	**Home**	1.54	2.46	4.84	5.07	13.97	13.91
	**0.350** **kg**	**Home**	1.55	2.81	5.29	5.70	15.44	15.35
	**0.450** **kg**	**Home**	1.68	2.79	5.03	6.94	16.50	16.44
	**Simple**	**Home**	1.88	2.76	4.61	4.49	13.89	13.74
	**Weak**	**Home**	0.65	1.01	2.13	3.36	7.28	7.15

The unit of normal force data is in Newton.
- The sum of the tangential forces of the index, middle, ring, little, and thumb should be equal to the weight of the handle. Table 7 shows the average tangential force produced by each finger and thumb during all conditions of the four experiments. The sum of the tangential forces produced by all the fingers and thumb was almost equal to the weight of the handle.

**Table 7:** Average tangential force data of the individual fingers and thumb during different conditions of the four experiments are shown.

Outcome variable	Condition	Thumb position	Index	Middle	Ring	Little	Thumb	I + M + R + L + T	Mass of the handle (N)	% of error
**Tangential force**	**Fixed**	**Home**	0.85	1	0.74	0.41	2.70	5.70	5.24	8.77
	**Free**	**Home**	0.77	1.08	1.25	1.14	1.09	5.33	5.24	1.71
	**Trapezoid**	**Top**	0.92	1.54	1.34	0.80	1.23	5.83	5.24	11.25
		**Home-Top**	0.82	1.30	1.28	1.45	1	5.85	5.24	11.64
	**Inverted Trapezoid**	**Home-Bottom**	0.62	1.19	1.50	1.31	1.15	5.77	5.24	10.11
		**Bottom**	0.43	0.70	1.13	2.66	0.93	5.85	5.24	11.64
	**0.150** **kg**	**Home**	0.77	1.10	1.64	2.03	1.15	6.69	5.88	13.77
	**0.250** **kg**	**Home**	0.82	1.31	1.92	2.54	1.19	7.78	6.86	13.41
	**0.350** **kg**	**Home**	0.89	1.50	2.26	2.80	1.15	8.60	7.84	9.69
	**0.450** **kg**	**Home**	1.04	1.73	2.52	3.22	1.15	9.66	8.82	9.52
	**Simple**	**Home**	0.79	1.54	1.99	2.06	1.22	7.62	6.86	11.07
	**Weak**	**Home**	0.68	1.16	1.74	2.32	1.22	7.14	6.86	4.08

The unit of tangential force data is in Newton.
- The sum of the moment due to the normal and tangential force of the individual fingers and thumb should be equal to zero. Table 8 shows the moment due to normal force (Mn) and tangential force (Mt) produced by each finger and thumb and the total moment at each thumb position during all conditions of the four experiments.

**Table 8:** Average moment due to normal and tangential force data of the individual fingers and thumb and total moment during different conditions of the four experiments are shown. Negative sign (‘−’) indicates clockwise moment.

Condition	Index		Middle		Ring		Little		Thumb		Total
	Mn	Mt	Mn	Mt	Mn (−)	Mt	Mn (−)	Mt	Mn	Mt (−)	(Mn + Mt)
**Fixed**	44.61	28.27	7.29	33	17.20	24.68	39.50	13.84	23.24	89.24	29
**Free**	43.59	25.51	8.18	35.65	40.89	41.39	103.15	37.70	28.16	36.09	40.06
**Trapezoid**	94.87	30.42	12.43	51.14	28.23	44.27	44.94	26.42	−110.88	40.82	34.67
	43.25	27.17	10.67	43.20	40.28	42.36	124.23	47.96	16.55	33.01	33.66
**Inverted Trapezoid**	54.95	20.61	9.58	39.56	46.91	49.69	103.57	43.49	31.69	37.98	61.13
	20.55	14.47	4.17	23.21	51.21	37.54	263.94	87.92	206.04	30.73	48.03
**0.150** **kg**	40.71	23.98	2.18	34.17	85.87	50.99	187.22	63.11	115.17	35.95	21.29
**0.250** **kg**	34.72	25.65	2.97	40.85	91.74	59.68	199.20	79	114.51	36.99	29.47
**0.350** **kg**	35.20	27.63	4.19	46.60	100.55	70.08	225.03	86.94	125.57	35.78	34.89
**0.450** **kg**	40.01	32.52	3.55	53.70	91.86	78.30	270.27	100	139.50	35.86	49.69
**Simple**	43.33	24.64	1.02	47.74	92.68	61.98	180.55	64.09	115.37	38.04	46.90
**Weak**	15.72	21.23	1.47	36.04	42.42	54.14	135.95	71.95	55.29	38.10	39.37

Mn represents moment due to normal force. Mn was computed by considering the center of pressure of the individual fingers. Mt represents moment due to tangential force. The unit of the Moment due to normal and tangential force data is in Newton millimetre.

### Experiment-4: Thumb normal force data validation

Since, in the fourth experiment, the thumb normal force data was shown as visual feedback to the participants for tracing the target force line, the root mean square error of the thumb normal force with reference to the target line was computed. The average root mean square error of the thumb normal force and standard deviation for the simple and weak grasp are 0.24 ± 0.05 N and 0.36 ± 0.07 N.

c) **Thumb displacement data validation**

Since the thumb displacement data collected during the experiments 2 and 3 was shown as visual feedback on the participant monitor to trace the pattern (in experiment 2) and target horizontal line (in experiment 3), root mean squared error was computed only for the displacement data of the second and third experiment.

#### Experiment 2

The root mean square error of the thumb displacement data with reference to the template pattern was computed for the first 100 datapoints of the static thumb positions (TOP, HOME-TOP, HOME-BOTTOM, BOTTOM). The average root mean square error of the thumb displacement data and standard deviation at four different thumb positions are 0.055 ± 0.016, 0.059 ± 0.023, 0.050 ± 0.008, 0.112 ± 0.033 cm.

#### Experiment 3

The root mean square error of the thumb displacement data with reference to the target line was computed. The average root mean square error of the thumb displacement data and standard deviation for the external loads of masses 0.150 kg, 0.250 kg, 0.350 kg and 0.450 kg was 0.021 ± 0.005, 0.024 ± 0.006, 0.024 ± 0.007 and 0.032 ± 0.018 cm.

### Behavioural validation

Only the right-hand dominant male volunteers were recruited for participation.

## Usage Notes

Grip width of the experimental handle used in experiments 1 and 2 = 6.6 cm

Grip width of the experimental handle used in experiment 3 and 4 = 6.2 cm

For all the experiments, the distance between the centers of individual finger sensors (excluding the thumb) = 2 cm

At the HOME position, the center of the thumb sensor was aligned midway between the middle and ring finger sensor centers for all the experiments

The thumb displacement data stored in the data file was obtained from the laser displacement sensor after calibration.

For experiment 1, the calibrated HOME position = 3.48 cm.

For experiment 2, the calibrated HOME position = 7 cm. Moving the thumb platform upwards by 1.5 cm would show a calibrated value of 5.5 cm. While translating the thumb platform downwards by 1.5 cm would show a calibrated value of 8.5 cm.

For experiment 3, the calibrated HOME position = 6.17 cm.

For experiment 4, the calibrated HOME position = 6.21 cm.

Moments for all the experiments were calculated with respect to the center of mass (COM) of the handle (represented as ‘X’). For the experimental handle of experiments 1 and 2, the center of the thumb sensor at HOME position lies at 0.6 cm below COM. In the case of the experimental handle used for the third and fourth experiment, the center of the thumb sensor at the HOME position lies 1 cm below COM.

The friction coefficient was computed by dividing the horizontal tangential force and normal force from when the platform translated until the finger slipped from the finger.

## Data Availability

The Matlab code provided with this article includes the code for fetching the fingertip force data of all the fingers and thumb displacement data from the dataset file. The nomenclature of the Matlab file is forceanddisp_dataplots.m. This code helps to average the normal forces, tangential forces of individual fingers separately and thumb displacement data across the trials and participants. Further, the code plots the averaged normal and tangential force data of the individual fingers and thumb. The Matlab software version used for this code was MATLABR2016b. We had also provided the code for preprocessing the dataset and it is named as filter_code.m. The Matlab code for the moment computation of the data collected from experimental handle-1 of the first experiment is given separately and it is named as momentcomputation.m. Matlab codes are available in figshare along with the raw dataset^21^.

## References

[1] Johansson RS, Westling G (1984). Roles of glabrous skin receptors and sensorimotor memory in automatic control of precision grip when lifting rougher or more slippery objects. Exp Brain Res.

[2] Winstein CJ, Abbs JH, Petashnick D (1991). Influences of object weight and instruction on grip force adjustments. Exp Brain Res.

[3] Zatsiorsky VM, Gao F, Latash ML (2003). Finger force vectors in multi-finger prehension. J Biomech.

[4] Zatsiorsky VM, Gao F, Latash ML (2003). Prehension synergies: Effects of object geometry and prescribed torques. Exp Brain Res.

[5] Santello M, Soechting JF (2000). Force synergies for multifingered grasping. Exp Brain Res.

[6] Aoki T, Latash ML, Zatsiorsky VM (2007). Adjustments to Local Friction in Multifinger Prehension. J Mot Behav.

[7] Cole KJ, Johansson RS (1993). Friction at the digit-object interface scales the sensorimotor transformation for grip responses to pulling loads. Exp Brain Res.

[8] Cadoret G, Smith AM (1996). Friction, not texture, dictates grip forces used during object manipulation. J Neurophysiol.

[9] Zatsiorsky VM, Gao F, Latash ML (2006). Prehension Stability: Experiments With Expanding and Contracting Handle. J Neurophysiol.

[10] Slota GP, Latash ML, Zatsiorsky VM (2012). Tangential Finger Forces Use Mechanical Advantage during Static Grasping. Journal of Applied Biomechanics.

[11] Solnik S, Zatsiorsky VM, Latash ML (2014). Internal Forces during Static Prehension: Effects of Age and Grasp Configuration. J Mot Behav.

[12] Rajakumar, B. & Skm, V. Comparable behaviour of ring and little fingers due to an artificial reduction in thumb contribution to hold objects. *PeerJ*, 10.7717/peerj.9962 (2020). 10.7717/peerj.9962 PMC7502246 32995096

[13] Banuvathy R, Varadhan SKM (2021). Distinct behavior of the little finger during the vertical translation of an unsteady thumb platform while grasping. Sci Rep.

[14] Marshall VC, Marshall RD (1963). Movements of the Thumb in Relation to Peripheral Nerve Injuries. Postgrad Med J.

[15] Kwakkel G (2017). Standardized measurement of sensorimotor recovery in stroke trials: Consensus-based core recommendations from the Stroke Recovery and Rehabilitation Roundtable. Int J Stroke.

[16] Eschmann H, Héroux ME, Cheetham JH, Potts S, Diong J (2019). Thumb and finger movement is reduced after stroke: An observational study. PLOS ONE.

[17] Nowak DA (2008). The impact of stroke on the performance of grasping: usefulness of kinetic and kinematic motion analysis. Neurosci Biobehav Rev.

[18] Savescu AV, Latash ML, Zatsiorsky VM (2008). A technique to determine friction at the finger tips. J Appl Biomech.

[19] Park J, Pažin N, Friedman J, Zatsiorsky VM, Latash ML (2014). Mechanical properties of the human hand digits: Age-related differences. Clinical Biomechanics.

[20] Rajakumar B, Dutta S, Varadhan SKM (2022). Support for mechanical advantage hypothesis of grasping cannot be explained only by task mechanics. Sci Rep.

[21] Rajakumar B, Varadhan, SKM (2022). figshare.

